# Iron‐dexamethasone nanoparticles mitigate acute neutrophilic inflammation

**DOI:** 10.1002/btm2.70173

**Published:** 2026-08-19

**Authors:** Michael L. Felder, M. Valentina Guevara, Daniel Kupor, Omolola Eniola‐Adefeso

**Affiliations:** ^1^ Department of Chemical Engineering University of Michigan Ann Arbor Michigan USA; ^2^ Department of Biomedical Engineering University of Illinois Chicago Chicago Illinois USA

**Keywords:** corticosteroid, drug delivery, nanoparticle, neutrophil, targeted

## Abstract

Neutrophils are the primary immune cell type present within the blood and the first to arrive at sites of inflammation. As such, neutrophils set the stage for how the inflammatory response progresses and resolves. In some cases of non‐resolving inflammation, excessive neutrophil accumulation can cause tissue damage. Accordingly, maintaining tight control over their recruitment and behavior at sites of inflammation is paramount. Historically, corticosteroids have been used to modulate inflammation, but they are associated with systemic side effects that have limited their use. Here, we developed an iron‐dexamethasone nanoparticle (DexOx NP) therapeutic that modulates neutrophil behavior and reroutes neutrophils away from sites of inflammation. DexOx NPs inhibit neutrophil activation, as indicated by prevention of L‐selectin shedding and reduced neutrophil extracellular trap formation. Furthermore, treatment with DexOx NPs in a murine acute lung injury model significantly reduced the total number of neutrophils in the bronchoalveolar lavage fluid while avoiding the side effects of systemic soluble dexamethasone phosphate delivery. Thus, DexOx NPs represent a new option to control non‐resolving neutrophilic inflammation.

**Translational Impact Statement**

This work introduces a targeted nanoparticle therapy composed entirely of dexamethasone that delivers the drug directly to overactive neutrophils, preventing excessive inflammation while avoiding harmful steroid side effects and unnecessary exposure to exogenous polymer materials. By redirecting and calming these “first responder” immune cells, our approach protects tissue from damage in severe inflammatory diseases such as acute lung injury. This strategy offers a more precise anti‐inflammatory treatment with lesser side effects than current steroid therapies.

## 1 INTRODUCTION

Neutrophils are the primary arm of the innate immune system, making up to 60% of circulating white blood cells in humans, and play a significant role in the progression of inflammation and the development of adaptive immune response.^1^ Following the onset of an acute inflammatory response, neutrophils are rapidly recruited to the site of inflammation.^2^, ^3^ Here, they activate their specific effector functions, such as phagocytosis, degranulation, or formation of neutrophil extracellular traps (NETs).^4^ These effector functions contain the invading pathogen and effectively remove it from the injury site. Additionally, neutrophils then set the stage for an expanded inflammatory response, where they interact and communicate with other immune cells like macrophages, lymphocytes, and dendritic cells to perpetuate and ultimately resolve inflammation.^5^ Subsequent resolution of inflammation is a tightly controlled process in which recruitment of infiltrating immune cells, like neutrophils, is stopped, and any remaining cells are removed from the tissue.^1^ However, non‐resolving inflammation can occasionally occur, resulting in tissue damage. Such non‐resolving inflammation commonly occurs in acute respiratory distress syndrome (ARDS).

ARDS is characterized by excessive infiltration of neutrophils into the lungs, leading to fluid and other blood cells within the alveolar space.^6^ ARDS can originate from different sources, with pneumonia or sepsis accounting for most cases.^7^ Current ARDS treatment strategies typically address the underlying cause of lung injury and include lung‐protective ventilation.^7^ Despite these measures, ARDS remains a severe condition with a mortality rate as high as 35%, affecting approximately 10% of all ICU patients.^8^, ^9^ Though steroids have historically shown poor effectiveness at moderating the inflammatory response in ARDS, a recent clinical trial has shown that systemic administration of soluble dexamethasone can improve outcomes in ARDS.^10^ However, these benefits are typically observed at high doses of dexamethasone with prolonged treatment regimens and appear to be limited to certain patient subgroups, where some patients experienced worse outcomes following treatment.^11^ As such, alternative methods to administer dexamethasone could prove beneficial for the treatment of ARDS.

Dexamethasone acts on cells via the glucocorticoid receptor, a ligand‐dependent transcriptional factor that resides in the cytoplasm of nearly all cells.^12^ The anti‐inflammatory effects of free dexamethasone on human neutrophil surface protein expression are known to be both time‐ and concentration‐dependent when treated in vitro, where extended incubation of dexamethasone with activated neutrophils led to a subsequent decrease in L‐selectin shedding.^13^ Furthermore, in a mouse lung injury model, dexamethasone prevents neutrophil migration into the inflamed lung tissue when delivered systemically.^14^ These anti‐inflammatory effects of dexamethasone also extend to cytokine production, where IL‐6 and TNF‐α were significantly reduced in ALI mice receiving dexamethasone.^15^ However, off‐target side effects, such as neutrophilia and depleted lymphocytes in the blood, are also associated with dexamethasone administration.^16^, ^17^ As such, targeted delivery mechanism dexamethasone to neutrophils could enhance its therapeutic effects while avoiding off‐target systemic effects. Particle‐based therapeutics are a promising option in this regard.

Particle therapeutics are known to interact with neutrophils in the bloodstream, where particles reroute neutrophils away from sites of inflammation.^18^, ^19^, ^20^, ^21^, ^22^ Particles that contain an active drug component within their particle matrix can have even further beneficial effects to modulate neutrophil function. Specifically, neutrophil phagocytosis of particles containing anti‐inflammatory drugs has a markedly greater effect on inhibiting neutrophil activation and migration than free drugs or cargo‐free particles.^23^, ^24^ From this, we see that particle internalization is a means by which neutrophil behavior can be altered.^18^ Dexamethasone‐loaded liposomes were used to target neutrophils in a sepsis model, and showed that targeted liposomes outperformed the free drug and successfully modulated neutrophil activation as measured by both total neutrophil accumulation at sites of inflammation, NETosis, and cytokine production.^25^ However, current formulations of particle‐based therapeutics are also associated with their own setbacks, including formulation stability, reproducibility, and drug loading.^26^, ^27^

In this work, we developed a stable dexamethasone nanoparticle (DexOx NP) formulation to target neutrophils and alleviate neutrophilic inflammation. DexOx NP were formed using a controlled nanoprecipitation and heating method. We first characterized the particles, assessing the impact of open‐air oxidation on particle surface properties and subsequent protein corona formation, and how these impacted interactions with human and mouse neutrophils. Ex vivo analysis using whole human blood and isolated human neutrophils showed DexOx NPs modulate neutrophil activation, as indicated by decreased L‐selectin shedding and reduced formation of NETs. In vivo, in a preclinical murine acute lung injury (ALI) model, DexOx NPs successfully attenuated neutrophilic inflammation while avoiding systemic effects typically associated with soluble systemic dexamethasone.

## 2 RESULTS AND DISCUSSION

### 2.1 Fabrication and physical characterization of Dex NP and DexOx NP

**FIGURE 1:**
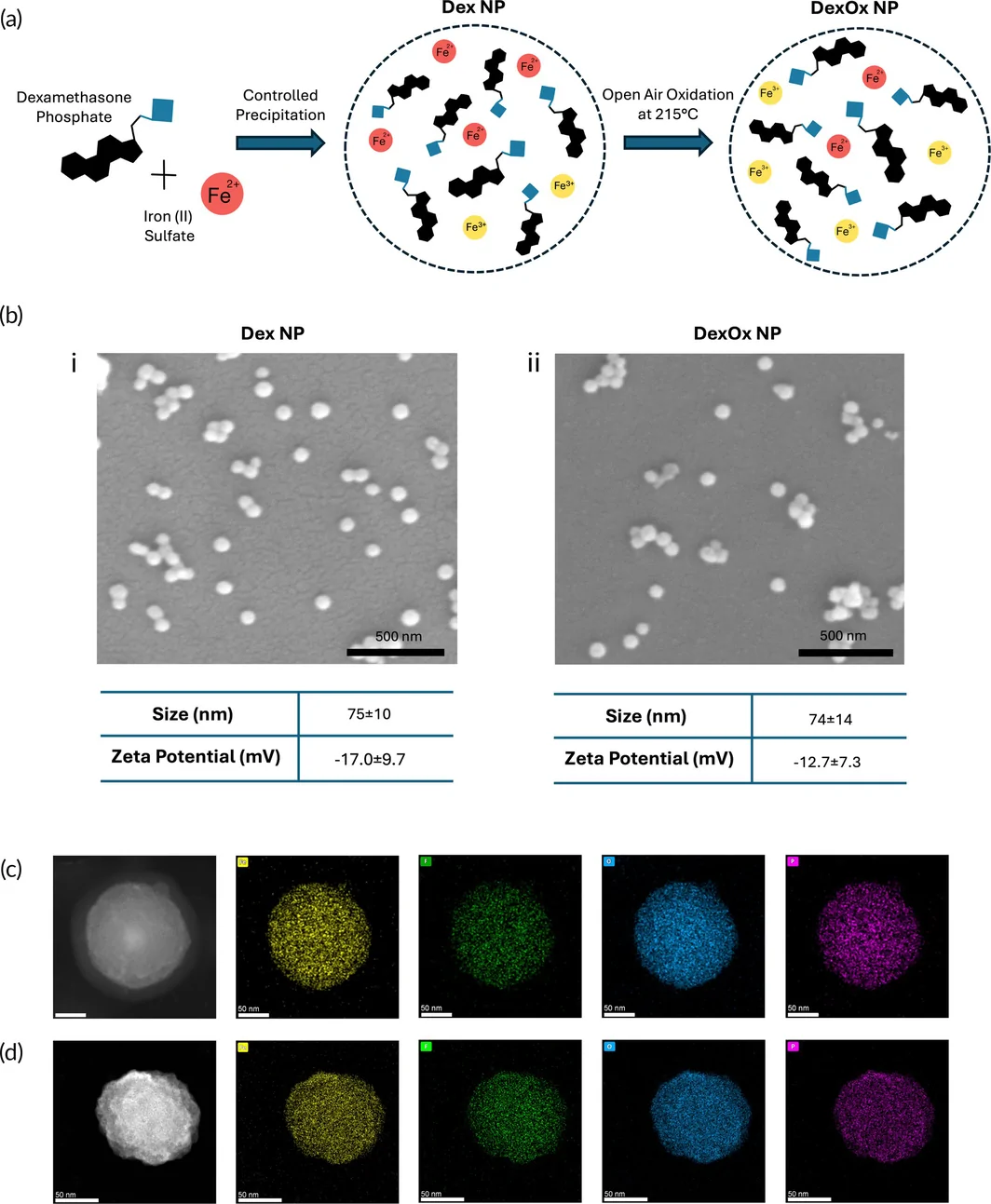
Fabrication and physical characterization of Dex NP and DexOx NP. (a) Fabrication protocol for Dex NP and DexOx NP; (b) SEM images depicting Dex NP (i) and DexOx NP (ii), where average diameter of particles before and after the heating step is approximately 75 nm. Scale bars are 500 nm. Zeta potential for both particles is between −10 and −20 mV, where no significant difference between Dex NP and DexOx NP is observed; (c) and (d) are STEM EDS maps for Dex NP (top) and DexOx NP (bottom), where even distribution of iron (yellow), fluorine (green), oxygen (blue), and phosphorus (magenta) indicate that iron and dexamethasone phosphate are throughout particle matrix. Scale bars are 50 nm.

To overcome limitations associated with current steroid drug delivery systems, we sought to develop a nanoparticle‐based platform that preserves drug stability and exhibits inherent targeting to specific cell types. To achieve this goal, we adapted a method that leverages transition metals to fabricate composite particles of steroid drugs.^28^ The particle fabrication process is depicted in Figure 1a, showcasing a nanoprecipitation method in which iron solution is added to dexamethasone phosphate solution, resulting in the formation of iron‐dexamethasone phosphate nanoparticles (Dex NP). Amorphous iron‐phosphate particle formation has been characterized in the literature, where an amorphous intermediate forms during ferrous phosphate fabrication, consistent with what we observe for Dex NP.^29^ These results suggest iron‐dexamethasone nanoparticle formation is due to a nucleation growth mechanism, where active dexamethasone is incorporated into the particle matrix. We next wanted to determine the therapeutic activity of Dex NP on neutrophils. However, these nanoparticles demonstrated limited association with neutrophils. Specifically, when FITC‐labeled Dex NP were incubated with human neutrophils in whole blood, no internalization was observed (Figure S1). Given that nanoparticle surface characteristics and the unique plasma protein corona formed when placed in whole blood govern cellular engagement and internalization,^30^ we sought to alter the iron‐dexamethasone phosphate nanoparticles. We hypothesized that ionic iron incorporated within the nanoparticle matrix could be leveraged to alter the surface chemistry and enhance interactions with neutrophils. In particular, neutrophils have been shown to readily internalize iron oxide nanoparticles,^31^ suggesting that oxidation of ionic iron in Dex NP could be used to form an iron oxide shell to boost Dex NP engagement with and uptake by neutrophils.

Of specific interest are dysopsonins, proteins that avoid clearance by phagocytes. Protein corona formation around iron oxide nanoparticles has been characterized, showing that both albumin and transferrin adsorbed poorly onto the surface of iron oxide nanoparticles.^32^ Albumin is a known dysopsonin.^33^ As such, we hypothesized that oxidation of the surface of the Dex NP, ergo the ionic iron present at the surface, could be a means to alter their characteristics and promote internalization by neutrophils. Iron oxidation of magnetite nanoparticles in open air has been shown to occur at temperatures as low as 200°C, where mixtures of iron oxides can form at the particle surfaces.^34^, ^35^, ^36^ To determine if heating Dex NP to facilitate iron oxide formation could be utilized, we heated Dex NP particles to 200, 215, and 230°C to see if any changes in surface protein adsorption occurred (Figure S2). Changes in surface protein composition occurred only at temperatures above 215°C, as no effect was observed for samples heated to 200°C. Based on this, we subjected the dexamethasone‐iron NPs to heating at 215°C to facilitate oxidation of the iron present on the particle surface.

Particles formed via the two‐step process (i.e., DexOx NPs) exhibited zeta potential consistent with that previously reported for iron oxide nanoparticles.^37^ Importantly, no significant change in diameter was observed, indicating no material was combusted from the particle surface with heating (Figure 1b,i‐ii). The absence of material loss was further confirmed via scanning transmission electron microscopy energy‐dispersive X‐ray spectroscopy (STEM–EDS), used to detect and quantify the relative amounts of carbon, oxygen, phosphorus, and fluorine within NPs before and after the heating step. EDS mapping (Figure 1c,d) revealed particles contain evenly distributed iron and dexamethasone phosphate, both before and after the oxidation step. XPS allowed us to characterize the top 10 nm of the particle surface and determine the materials present and their oxidation states. Specifically, we sought to gain insight into the oxidation state of iron. If the open‐air heating step formed iron oxide, we would expect to see a shift from Fe^2+^ to Fe^3+^. XPS surface analysis revealed a shift toward Fe^3+^ at the particle surface after heating, consistent with iron oxide formation (Figure S3A–D).

We used FTIR analysis to further characterize the particle matrix and confirm the presence of dexamethasone phosphate loaded into the particle (Figure S3E). Prior work looking at polymers loaded with dexamethasone has shown that heating dexamethasone‐containing materials to temperatures as high as 200°C for 1 h resulted in just over 60% recovery of dexamethasone, a length of time far longer than the minutes our particles are oxidized in open air.^38^ Furthermore, thermogravimetric studies on dexamethasone heat stability have shown dexamethasone to undergo thermal degradation only at temperatures higher than 200°C, with the onset of material deterioration occurring at 256°C, when heated at rates of 5 or 10°C per minute.^39^, ^40^ Interestingly, we found that major peaks were conserved before and after heating, indicating that no significant degradation of dexamethasone phosphate occurred during the oxidation step. To confirm that dexamethasone could be released from the DexOx NP matrix and remain therapeutically active, we performed ELISA on the supernatant from DexOx NP incubated in phosphate‐buffered saline (PBS) under physiological conditions for up to 8 h. We found that dexamethasone released from DexOx NP maintains activity comparable to free dexamethasone in solution (Figure S4). These data indicate that the dexamethasone contained within the DexOx NP is not compromised during the iron oxidation process. However, if any oxidative damage were done to dexamethasone present within the DexOx NP, it would likely be composed of 17‐Oxo dexamethasone, a known product of dexamethasone oxidation.^38^, ^41^ Overall, these results suggest that iron oxide likely forms on the particle surface of DexOx NP while retaining active dexamethasone phosphate within the particle matrix.

### 2.2 Particle characterization in biological environments

**FIGURE 2:**
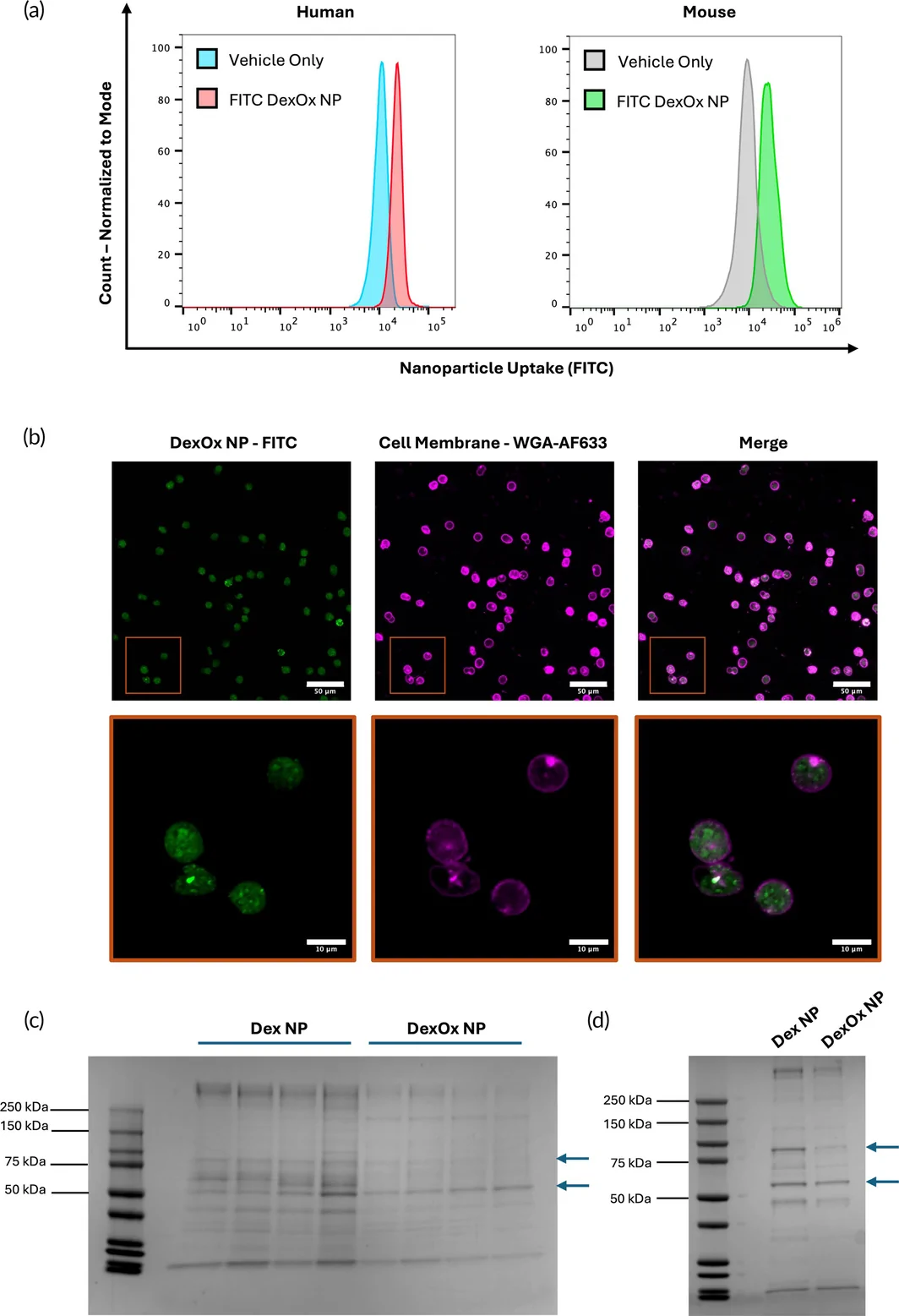
Protein adsorption onto Dex NP and DexOx NP surface and particle‐neutrophil interactions. (a) Shift in peak for neutrophils that received FITC‐labeled DexOx NP is observed for both human and mouse neutrophils; (b) confocal images of isolated human neutrophils that received FITC‐labeled DexOx NP and WGA‐Alexa Fluor 633‐labeled neutrophils. Scale bars are (top) 50 μm and (bottom) 10 μm; (c) protein adsorption onto particle surfaces using plasma from 4 independent human donors and (d) pooled plasma from 5 healthy BALB/C mice.

We next sought to fully characterize the biological activities of DexOx NPs and their cellular uptake. To assess particle‐cell interactions, we labeled both Dex NP and DexOx NP with FITC to evaluate their association with neutrophils ex vivo in both human and mouse whole blood samples. Flow cytometry analysis for human samples reveals a distinct shift in FITC signal for whole blood treated with labeled DexOx NP (Figure 2a), indicating nanoparticle association with neutrophils. Similarly, we observed a distinct shift in the neutrophil population for mouse whole blood treated with FITC‐labeled DexOx NP (Figure 2a). To determine whether DexOx NP were internalized by neutrophils, we treated isolated human neutrophils with FITC‐labeled DexOx NP (green) and stained cell membranes with WGA‐AF633 (magenta). Confocal images (Figure 2b) showed internalization of DexOx NP by neutrophils, which may contribute to enhanced drug efficacy when used in inflammatory conditions.

Next, we wanted to confirm the impact of particle oxidation on protein corona formation on DexOx NPs. Total proteins adsorbed to the particle surface decreased for particles that under went oxidation, with incubation in both human (Figure 2c) and mouse (Figure 2d) plasma. Furthermore, there is a distinct decrease in the protein bands at ~60 kDa and ~80 kDa corresponding to albumin and transferrin—proteins known to adsorb poorly to iron oxide surfaces.^32^ As albumin is the most abundant protein present in plasma and known to inhibit internalization of particles when present on the particle surface, we hypothesize that lower amounts of albumin are what led to increased particle internalization for the DexOx NP. Further, there is a slight increase in band intensity between 150 and 200 kDa for human donors, which aligns with various immunoglobulins present in the blood^42^ that are known to activate the complement system and drive phagocytosis.^43^, ^44^ Thus, the increase in these immunoglobulins on the surface of DexOx NP could aid immune recognition, facilitating their internalization by neutrophils.

### 2.3 Evaluate hemocompatibility in blood and anti‐inflammatory activities on neutrophils in simulated acute inflammation

DexOx NP internalization by neutrophils highlights their potential as a targeted drug delivery system, so we next assessed their safety in whole human blood. Our red blood cell (RBC) studies confirmed that DexOx NP did not induce hemolysis (Figure S5A), nor did they activate platelets (Figure S5B). Additionally, DexOx NP had no effect on L‐selectin expression in unactivated neutrophils, indicating no unintended neutrophil activation (Figure S6A). Altogether, these findings indicate that DexOx NPs can be delivered systemically.

**FIGURE 3:**
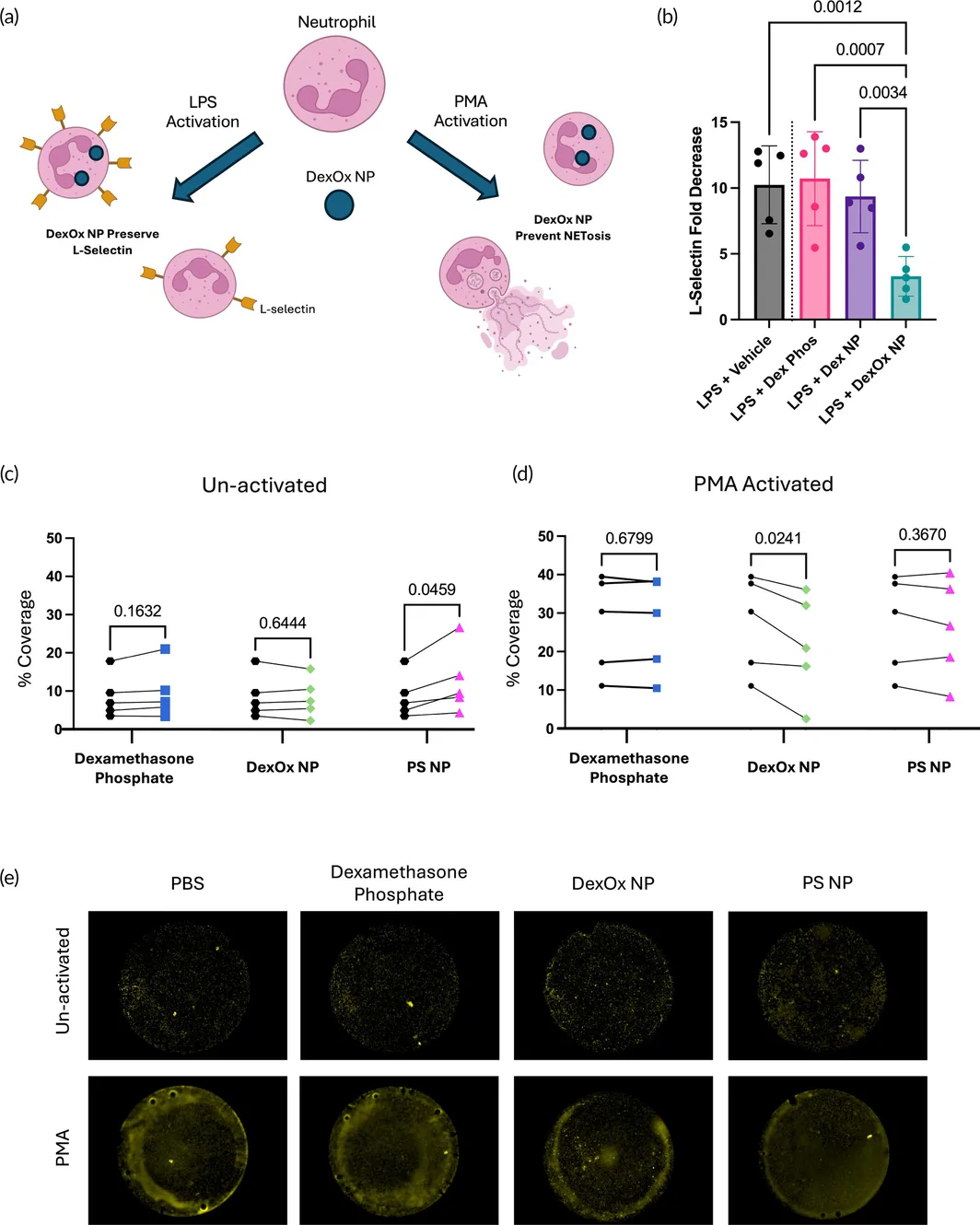
Ex vivo interactions of dexamethasone treatments with human neutrophils. (a) Schematic of in vitro analysis conducted on human neutrophils; (b) DexOx NP significantly preserved L‐selectin in LPS‐activated neutrophils as compared to free dexamethasone phosphate and Dex NP. Data points represent individual human donors. Bars show average L‐selectin fold decrease and error bars represent standard error; (c) DexOx NPs do not induce any additional NETosis in unactivated neutrophils as compared to unactivated control. Data points represent individual human donors; (d) DexOx NP significantly reduced the total amount of NETs formed as compared to a PMA‐only control. Data points represent individual human donors. (e) Representative images of wells for each of the groups tested, where the yellow color represents NETs. Statistical analysis was conducted using a one‐way ANOVA with post‐hoc Fisher's LSD test for (B). Paired *t*‐tests were performed for each treatment group as compared to the untreated control for (c) and (d). Figure 3a was made using BioRender.com.

Prior work in our lab has shown that particle internalization can improve the activity of anti‐inflammatory molecules.^23^ Since the glucocorticoid receptor is in the cytosol of the cell, it likely has more rapid activation from internalized particles as opposed to a freely solubilized drug that has to diffuse into the interior of the cell. Given their neutrophil association and ability to release active dexamethasone, we evaluated the ability of DexOx NP to prevent neutrophil activation in the presence of lipopolysaccharide (LPS) and phorbol 12‐myristate 13‐acetate (PMA) (Figure 3a). LPS is known to induce characteristic changes associated with neutrophil activation, such as L‐selectin shedding. DexOx NP significantly reduced L‐selectin shedding by 68% (*p* = 0.0012) in LPS‐activated neutrophils (Figure 3b), consistent with a previous report of a similar impact of dexamethasone treatment on neutrophils exposed to platelet‐activating factor.^13^ Furthermore, we show DexOx NPs have significantly improved anti‐inflammatory activity over free dexamethasone phosphate (*p* = 0.0007) or poorly internalized Dex NP (*p* = 0.0034), where neither dexamethasone phosphate nor Dex NP induced any changes in L‐selectin expression for LPS‐activated neutrophils. This enhanced effect of DexOx NP may be attributed to localized delivery of dexamethasone inside neutrophils, enabling direct interaction with the glucocorticoid receptor, as confirmed by the mitigating effect of mifepristone (Figure S6B).

To confirm anti‐inflammatory impact of DexOx NP on neutrophils is linked to the active dexamethasone within the particle matrix and not the iron oxide on the surface, we also evaluated the effect of iron oxide nanoparticles on L‐selectin shedding in LPS‐activated neutrophils. We used open‐air oxidation and heating to facilitate the production of Fe_2_O_3_ NP on commercially acquired Fe_3_O_4_ iron oxide nanoparticles. These two particle groups allowed us to discern the impacts of either Fe^2+^ or Fe^3+^ on neutrophil activation. We found that both Fe_2_O_3_ and Fe_3_O_4_ nanoparticles had no effect on L‐selectin preservation in neutrophils (Figure S7). These results confirm that the anti‐inflammatory activity of DexOx NP is driven by released dexamethasone rather than the iron present within the particle matrix.

Next, we evaluated the impact of DexOx NPs on NETosis, a process in which activated neutrophils expel their intracellular contents and DNA to contain materials at the site of infection,^45^ and also drives inflammation in diseases such as ARDS.^46^ Accordingly, maintaining tight control over NETosis is crucial for maintaining a proper immune response to invading pathogens. Corticosteroids are shown to inhibit p47^phox^ subunit of NADPH, an enzyme central to neutrophil production of reactive oxygen species and NET formation.^47^, ^48^ Isolated neutrophils were treated with dexamethasone phosphate solution, DexOx NPs, or polystyrene particles, activated with PMA, and monitored over 5 h for extracellular DNA release via SytoxGreen staining.^24^ We opted to use 100 nm polystyrene particles as a cargo‐free control to help ascertain the impacts of particle internalization versus particles loaded with active therapeutic. DexOx NPs did not induce NETosis in unactivated neutrophils (Figure 3c), consistent with their anti‐inflammatory profile. Importantly, DexOx NPs significantly reduced NETosis by 21% (*p* = 0.0241) in PMA‐activated neutrophils (Figure 3d), whereas free dexamethasone phosphate, Dex NP (Figure S8), and polystyrene particle controls had no effect. Representative images of NETs formed during each treatment are shown in yellow (Figure 3e). Our particles were shown to significantly decrease the total number of NETs formed in PMA‐activated neutrophils, likely due to inhibition of NADPH oxidase (NOX), where NETosis starts by activation of the NOX complex.^45^ Thus, utilization of our corticosteroid nanoparticles, and specifically dexamethasone, is a means by which NETosis can be mitigated in a targeted manner.

### 2.4 In vivo activity of DexOx NP in a murine acute lung injury model

We next aimed to understand how DexOx NP would perform in vivo in an ALI model. Initial toxicity studies were conducted on healthy BALB/c mice. We explored the impacts of particle types on organ injury, where we analyzed levels of AST in uninjured mice over the course of 7 days (Figure S9A). DexOx NPs did not induce any significant changes in liver enzyme levels as compared to the vehicle‐only control. Furthermore, no significant changes in total leukocyte counts (Figure S9B,C) or total weight (Figure S9D) occurred following administration of DexOx NP.

**FIGURE 4:**
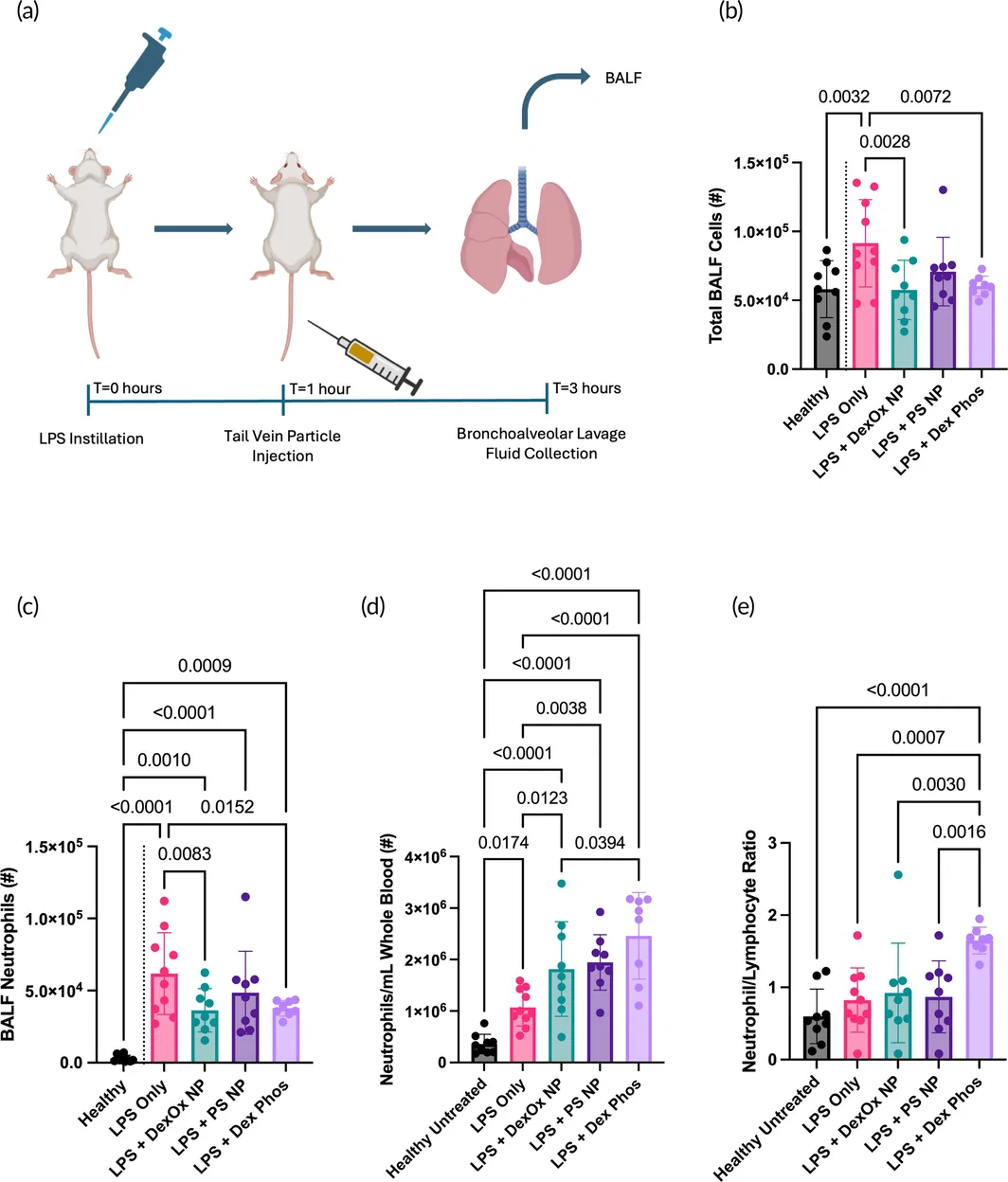
In vivo activity of DexOx NP in acute lung injury model. (a) Diagram depicting acute lung injury model instillation and treatment timeline; (b) total number of infiltrated immune cells found in the bronchoalveolar lavage fluid (BALF) for all treatment groups; (c) total number of infiltrated neutrophils in the bronchoalveolar lavage fluid (BALF) for all treatment groups; (d) total number of neutrophils per mL of whole blood; (e) Lymphocyte to neutrophil ratio in whole blood. Data points represent individual mice, and error bars represent standard error. Statistical analysis was conducted using a one‐way ANOVA with post‐hoc Fisher's LSD test for (b)–(e). Outliers were calculated using a ROUT test. Figure 4a was made using BioRender.com

Next, we evaluate the ability of DexOx NPs to reduce injury caused by inflammatory neutrophils in vivo in an LPS‐induced (ALI) model (Figure 4a). It is well documented that particle‐based therapeutics divert neutrophils away from sites of inflammation in vivo in mice with ARDS.^18^, ^22^, ^23^ Here, we included drug‐free 100 nm polystyrene nanoparticles as a non‐therapeutic control that are also phagocytosed by mouse neutrophils similar to DexOx NPs (Figure S10).

Particles were administered via tail vein injection 1 h after LPS injury induction, and mice were euthanized 2 h later for bronchoalveolar lavage fluid (BALF) and whole blood collection. Neutrophil transmigration into the lungs, an indication of lung injury severity, was used to assess therapeutic effectiveness.^49^ Both DexOx NP and free dexamethasone significantly reduced the total number of infiltrated immune cells by 37% (*p* = 0.0028) and 34% (*p* = 0.0072) as compared to the LPS‐only control, respectively (Figure 4b). Neutrophil counts in the BALF were also reduced by 41% (*p* = 0.0083) for DexOx NP and by 39% (*p* = 0.0152) for free dexamethasone compared to the LPS‐only control (Figure 4c). In contrast, cargo‐free polystyrene particles had no effect on total BALF cells or neutrophils as compared to the LPS‐only control. No change in BALF macrophage counts was observed across LPS‐treated groups (Figure S11B), highlighting the neutrophil‐targeted effect of DexOx NP through dexamethasone delivery.

We utilized ELISAs to quantify cytokines and chemokines in the BALF to confirm the anti‐inflammatory effects of treatments. Both DexOx NP and free dexamethasone significantly reduced BALF IL‐6 and TNF‐α as compared to both the LPS‐only and cargo‐free particle controls (Figure S12A,B). Additionally, DexOx NPs significantly reduced KC—a chemokine with a direct role in attracting neutrophils to sites of inflammation, while free dexamethasone significantly reduced both MIP‐1 and KC as compared to the cargo‐free particle control (Figure S12C,D).

Seeing as both DexOx NP and free dexamethasone significantly altered BALF cellular composition and soluble inflammation markers in vivo, we assessed the impact of both treatments on systemic side effects. Intravenous free dexamethasone is known to induce neutrophilia in the blood and reduce lymphocyte counts.^16^ We therefore evaluated whether DexOx NP could moderate neutrophil infiltration into inflamed tissue while avoiding characteristic systemic effects of freely administered dexamethasone. As shown in Figure 4d, free dexamethasone significantly increased blood neutrophil counts compared to both the LPS‐only control and DexOx NP by 57% (*p*< 0.0001) and 26% (*p* = 0.0394), respectively. While DexOx NP caused a 41% (*p* = 0.0123) increase in neutrophil counts versus LPS only, levels were similar to those seen with polystyrene NP, suggesting that the moderate increase in neutrophil count was likely associated with particle administration and less with dexamethasone release from the particle matrix. Importantly, only free dexamethasone significantly increased the neutrophil‐to‐lymphocyte ratio compared to all other treatment groups (Figure 4e), with a 64% (*p*< 0.0001) increase compared with the LPS‐only control. This is consistent with its known systemic side effects, yet the effect was not observed with the DexOx NP.

## 3 CONCLUSIONS

Corticosteroids are known immune modulators and have been used for decades to treat numerous inflammatory diseases.^50^ However, their use is restricted due to the number and severity of adverse side effects, where this class of medications can only be taken for limited periods of time.^50^, ^51^ As such, finding alternative delivery methods for corticosteroids, specifically targeted therapies, could improve their effectiveness and widen their scope of use. Our work aimed to develop a particle‐based method of corticosteroid delivery, where our new formulation enables the controlled release of dexamethasone directly to neutrophils involved in acute inflammation. DexOx NPs represent a dexamethasone formulation that enables direct drug delivery to neutrophils in systemic circulation. We demonstrated that the change in particle behavior with DexOx NPs had to do with the altered protein corona that formed once particles were administered. Moreover, these particles exhibit potent anti‐inflammatory activity, reducing L‐selectin shedding, preventing NET formation in activated neutrophils, and mitigating lung inflammation and injury in an ALI model. Finally, DexOx NPs improved outcomes in an ALI model without inducing the systemic neutrophilia or lymphopenia associated with free dexamethasone phosphate administration.

## 4 MATERIALS AND METHODS

All materials were purchased from MilliporeSigma without any further purification unless otherwise stated.

### 4.1 Study approvals

Human blood was collected from healthy male and female donors aged 20–30 years old under a protocol approved by the University of Michigan Internal Review Board (IRB: #HUM00013973). Informed and written consent was obtained from each donor prior to donation. All donors were compensated for their participation. Sex differences were not accounted for in this study.

Animal studies were completed under a protocol approved by the University of Michigan Institutional Animal Care and Use Committee (#PRO00010572) and followed additional guidelines set by the National Institute of Health. 7–8 week old BALB/c mice were purchased from Jackson Laboratories and kept in pathogen‐free housing for the duration of study.

### 4.2 Particle fabrication

50 mg of dexamethasone phosphate disodium salt (Catalog # D1159‐5G) was dissolved in 10 mL water and stirred at 600 rpm in an open beaker. 10 mg of iron sulfate heptahydrate (Catalog # F8633‐250G) was dissolved in 50 μL of water and added dropwise to the stirring dexamethasone phosphate solution. Stirring continued for 10 min, and particles were collected via centrifugation and freeze‐dried using a Labconco lyophilizer (Dex NP). Freeze‐dried particles were briefly heated at 200, 215, or 230°C to facilitate iron oxidation within the particle matrix (DexOx NP). Fluorescein (FITC)‐loaded DexOx NP were fabricated in the same manner as unloaded particles, whereby 1 mg FITC was included in dexamethasone phosphate solution, with all other fabrication parameters kept constant, and were heated to 215°C to facilitate oxidation.

### 4.3 Physical and chemical characterization

Scanning electron microscopy (SEM) was completed using a JEOL 7800FLV. Transmission electron microscopy (TEM) and energy dispersive spectroscopy (EDS) were completed using a Thermo Fisher Talos F200X G2. Powder X‐Ray Diffraction (XRD) was completed using a Rigaku Ultima IV system. Fourier transform infrared spectroscopy (FTIR) was completed using a Shimadzu IRTracer‐100 Spectrophotometer. X‐ray photoelectron spectroscopy (XPS) was completed using a Kratos Axis Supra+.

### 4.4 In vitro and ex vivo characterization

*Dexamethasone release assay*—DexOx NP were suspended in PBS at a concentration of 1 mg/mL and continuously rotated end‐over‐end at 37°C. At predetermined timepoints (*T* = 1, 2, 4, and 8 h) samples were centrifuged to pellet particles, and supernatant was removed and flash frozen before storing supernatant at −80°C. Supernatant was replaced with fresh PBS, particles were resuspended in solution, and samples were returned to 37°C incubator until next time point. Active dexamethasone present within degradation samples was analyzed using a dexamethasone ELISA kit (MyBioSource; Catalog # MBS2548580).

*Platelet activation*—Heparinized whole blood was centrifuged at 200 g for 15 min immediately following collection, and platelet‐rich plasma (PRP) was collected as the upper fraction of separated blood. 100 μL PRP per sample was aliquoted prior to the addition of 0.05 mg/mL, 0.1 mg/mL, or 0.5 mg/mL final diluted concentration of dexamethasone phosphate, Dex NP, or FeO Dex NP. Adenosine diphosphate was used as a positive control for platelet activation. Platelets were incubated at 37°C for 2 h before staining with anti‐CD41/61 and anti‐CD62P as platelet identification and platelet activation markers, respectively. Samples were fixed using 1‐step Fix/Lyse (Invitrogen; Catalog # 00‐5333‐57) before cleaning via centrifugation and resuspended in FACS buffer. Sample analysis was completed using an Attune NxT flow cytometer.

*Red blood cell lysis*—Heparinized whole blood was centrifuged at 2250 g for 15 min immediately following collection, and red blood cells (RBCs) were collected as the lower fraction of separated blood. Plasma fraction of blood was removed, and RBCs were resuspended in an equivalent volume of PBS. 100 μL RBC suspension was aliquoted prior to the addition of 0.05 mg/mL, 0.1 mg/mL, or 0.5 mg/mL final diluted concentration of dexamethasone phosphate, Dex NP, or FeO Dex NP. 5% Triton‐X was added to positive control RBC lysis samples. RBCs were incubated at 37°C for 2 h before samples were resuspended in 500 μL PBS and centrifuged to collect supernatant. Supernatant absorbance was analyzed at 415 nm using a Synergy H1 Microplate Reader, and absorbance was associated with RBC lysis.

*Particle internalization by neutrophils*—Heparinized whole blood was collected from healthy human donors. Neutrophil isolation was completed using a stacked density gradient with Histopaque‐1119 (Catalog # 11191‐100ML) and Ficoll‐Paque PLUS (Cytiva; Catalog # 17144002), where 4 mL Histopaque was first pipetted into a 15 mL conical tube, followed by 4 mL Lymphoprep, and finally 5 mL whole blood was gently pipetted onto Lymphoprep. Density gradient columns were centrifuged at 1440 g for 20 min, and the top layers of plasma, peripheral blood mononuclear cells (PBMCs), and Lymphoprep were removed. The light pink layer separating the Histopaque and Lymphoprep layers was collected, as it contained isolated neutrophils. Neutrophils were counted and resuspended at a concentration of 50,000 neutrophils per mL HBSS. 450 μL isolated neutrophil suspension was aliquoted per well in a 96‐well plate coated with Poly‐L‐Lysine to facilitate neutrophil adhesion to the surface. Unadhered neutrophils were washed, and 0.1 mg/mL final diluted concentration of either FITC‐loaded Dex NP or FITC‐loaded FeO Dex NP was incubated with neutrophils for 30 min. Unadhered particles were washed, and cells were fixed using 1‐Step Fix/Lyse (Invitrogen; Catalog # 00‐5333‐57) prior to cell membrane staining using Wheat Germ Agglutinin (WGA) labeled with Alexa Fluor 633. Residual WGA was washed from samples, and then imaged on a Nikon A1S confocal microscope. Particle phagocytosis was confirmed by visualization of fluorescent particles within the neutrophil membrane.

*Human protein adsorption onto particle surface*—Heparinized whole blood was collected from healthy human donors and centrifuged at 2250 g for 15 min, where the upper clear fraction constituted plasma. Both Dex NPs and DexOx NPs were suspended in 1 mL plasma at a concentration of 0.1 mg/mL and incubated on a rotator at 37°C for 5 min. Samples were centrifuged at 21,000 g for 10 min to pellet particles and subsequently washed 3 times with PBS to remove any free plasma proteins. Particles were resuspended in 50 μL non‐reducing buffer (Thermo Fisher; Catalog # 39001) and heated to 100°C for 5 min and then cooled to 4°C. Particle/protein suspension was then centrifuged at 21,000 g for 10 min to pellet particles, and transferred to gel (Invitrogen; Catalog # XP04200BOX) and run at 200 volts alongside a SDS molecular weight ladder (Bio‐Rad; Catalog # 1610374) for 30 min. Gels were stained overnight with Coomassie Blue (Life Technologies; Catalog # LC6065) before washing with DI water and imaging.

*Mouse protein adsorption onto particle surface*—Heparinized whole blood was collected from mice via cardiac puncture and centrifuged at 2250 g for 15 min, where the upper clear fraction constituted plasma. Plasma was pooled from 5 mice. Both Dex NPs and DexOx NPs were suspended in 1 mL plasma at a concentration of 0.1 mg/mL and incubated on a rotator at 37°C for 5 min. Samples were centrifuged at 21,000 g for 10 min to pellet particles and subsequently washed 3 times with PBS to remove any free plasma proteins. Particles were resuspended in 50 μL non‐reducing buffer (Thermo Fisher; Catalog # 39001) and heated to 100°C for 5 min and then cooled to 4°C. Particle/protein suspension was then centrifuged at 21,000 g for 10 min to pellet particles, and transferred to gel (Invitrogen; Catalog # XP04200BOX) and run at 200 volts alongside a SDS molecular weight ladder (Bio‐Rad; Catalog # 1610374) for 30 min. Gels were stained overnight with Coomassie Blue (Life Technologies; Catalog # LC6065) before washing with DI water and imaging.

*Neutrophil activation and dexamethasone treatments*—Heparinized whole blood was collected from healthy human donors. 100 μL whole blood was added to individual tubes, and activated samples received lipopolysaccharide for 30 min. Following activation, treatment groups were added at a final concentration of 0.1 mg/mL and incubated for an additional 2 h prior to cell staining and fixation. Cells were stained with anti‐CD11b (BioLegend; APC) and anti‐CD62L (BioLegend; FITC) for 30 min on ice before addition of 2 mL Fix/Lyse (Invitrogen; Catalog # 00‐5333‐57). Samples were cleaned and resuspended in FACS buffer before running on an Attune NXT flow cytometer. Mifepristone was added to select samples to better understand the role of the glucocorticoid receptor on particle activity. Briefly, 10 μM mifepristone was added to whole blood 30 min prior to administration of DexOx NP, both with and without LPS activation. Cells were incubated for an additional 2 h before flow cytometry as previously described.

*NETosis and dexamethasone treatments*—Heparinized whole blood was collected from healthy human donors. Neutrophil isolation was completed using a density gradient, where whole blood was layered onto Ficoll Plus (1.077) and centrifuged at 1440 g for 20 min. The top (plasma) and bottom (RBCs and neutrophils) were collected into separate tubes. Dextran sedimentation was used to separate RBCs and neutrophils. Residual RBCs in isolated neutrophils were removed via hypotonic/hypertonic saline additions. Neutrophils were plated at a density of 1 × 10^5^ neutrophils/well into poly‐L‐lysine (Catalog # P4704‐50 mL) coated 96‐well plates and co‐incubated with designated treatments at 37°C, 5% CO_2_, and >80% humidity for 1 h to allow neutrophils to internalize treatments and adhere to the plate surface. Unattached neutrophils were removed, and standard media or PMA (20 nM) activating media were added to designated wells, alongside SytoxGreen (Invitrogen; Catalog # S7020) for NET visualization. Wells were imaged using an EVOS M7000 on the GFP channel every hour for 5 h, and % Coverage was calculated using MatLab image analysis software, consistent with previously described methods.^24^

### 4.5 In vivo characterization

*Toxicity in healthy mice—Healthy* 8–10 week‐old BALB/c mice were treated with dexamethasone phosphate, Dex NP, or DexOx NP at concentrations of either 5 mg/kg or 10 mg/kg, where treatments were administered via tail vein injection in PBS. Mice were weighed daily for 1 week post treatment administration. 1 week post treatment administration, animals were euthanized via carbon dioxide asphyxiation, and whole blood was collected via cardiac puncture, where heparin was used as the anti‐coagulant. Whole blood samples were collected and stained using CD45 (BioLegend; Brilliant Violet 711), CD11b (BioLegend; FITC), Ly6G (BioLegend; APC), and Ly6C (BioLegend; Brilliant Violet 421) to determine total neutrophil and monocyte counts via flow cytometry, and then whole blood was centrifuged at 2250 g for 15 min to collect the plasma fraction. Plasma was then analyzed for aspartate aminotransferase (AST) to understand the impact of treatment administration on liver toxicity. AST was measured using an ELISA (Catalog # MAK467‐1KT), and treatment groups were compared to an untreated healthy control.

*LPS induced ali model—*BALC/c mice were briefly anesthetized using isofluorane, and LPS (50 μL; 0.4 mg/mL PBS) was instilled orotracheally. 1 h post‐instillation, treatment groups were administered via tail vein injection in PBS, where particle groups were treated at a 10 mg/kg dose, and dexamethasone phosphate was treated at a 0.323 mg/kg dose. The dexamethasone phosphate dose was based on a clinical dosing scheme for acute respiratory distress syndrome, calculated using the dose administered to patients (20 mg) and average human mass (62 kg).^10^, ^52^ 2 h post‐treatment, mice were euthanized via carbon dioxide asphyxiation, and blood was collected via cardiac puncture. Bronchoalveolar lavage fluid (BALF) was collected by flushing lungs with PBS 3 times. BALF was centrifuged at 500 g for 5 min to collect supernatant for ELISAs, and BALF cells were collected to determine total cell counts and cell composition via hemacytometer using Turk Blood Diluting Fluid (Fisher Sci; Catalog # 8850‐32) and flow cytometry, respectively. BALF cells were stained using CD45 (BioLegend; Brilliant Violet 711), CD11b (BioLegend; FITC), Ly6G (BioLegend; APC), and CD11c (BioLegend; Brilliant Violet 421) to determine total neutrophil and macrophage compositions. Whole blood samples were stained using CD45 (BioLegend; Brilliant Violet 711), CD11b (BioLegend; FITC), Ly6G (BioLegend; APC), and Ly6C (BioLegend; Brilliant Violet 421) to determine total neutrophil and monocyte compositions via flow cytometry. Total white blood cell counts were determined using a hemacytometer and Turk Blood Diluting Fluid (Fisher Sci; Catalog # 8850‐32).

*ELISA*s—IL‐6 ELISA (Invitrogen; Catalog # 88‐7064‐88) was run according to the manufacturer's instructions to determine the total concentration of the corresponding IL‐6 within the ALI BALF. TNF‐α, KC, and MIP‐2 ELISAs were analyzed using the Immune Monitoring Core at the University of Michigan.

## AUTHOR CONTRIBUTIONS

**M. Valentina Guevara:** Investigation; writing, review and editing. **Omolola Eniola‐Adefeso:** Conceptualization; funding acquisition; writing – review and editing; supervision. **Daniel Kupor:** Investigation; writing – review and editing. **Michael L. Felder:** Conceptualization; investigation; writing – original draft; validation; writing – review and editing; formal analysis.

## FUNDING INFORMATION

This work was funded in part by NIH R01 HL145709 (O.E.‐A.) and NIH T32 GM145304 (M.L.F).

## CONFLICT OF INTEREST STATEMENT

M.L.F., D.K., and O.E.A. have filed a patent titled “Composite drug particles and uses thereof” (US Patent Number: US20240165130A1). The other authors declare no competing interests.

## Supporting information


**Figure S1:** Dex NP particle associations with human neutrophils in whole blood.
**Figure S2:** Surface protein corona around Dex NP heated to various temperatures.
**Figure S3:** Physical characterization of Dex NP and DexOx NP.
**Figure S4:** DexOx NP degradation In PBS.
**Figure S5:** Dex NP and DexOx NP safety characterization in whole human blood.
**Figure S6:** Characterization of human neutrophil activation with dexamethasone treatments.
**Figure S7:** Human neutrophil activation with iron oxide nanoparticle treatments.
**Figure S8:** NET formation following incubation of human neutrophils with Dex NP.
**Figure S9:** In vivo safety of dexamethasone treatments.
**Figure S10:** Polystyrene nanoparticle associations with mouse neutrophils.
**Figure S11:** In vivo characterization of DexOx NP behavior as compared to free dexamethasone and polystyrene NP.
**Figure S12:** Mouse BALF cytokine and chemokine analysis.

## Data Availability

The data that support the findings of this study are available in the supplementary material of this article.

## References

[1] Fullerton JN , Gilroy DW . Resolution of inflammation: a new therapeutic frontier. Nat Rev Drug Discov. 2016;15:551‐567. 27020098 10.1038/nrd.2016.39

[2] El Brihi J , Pathak S . Normal and abnormal complete blood count with differential. StatPearls. StatPearls Publishing; 2025. 38861622

[3] Germolec DR , Shipkowski KA , Frawley RP , Evans E . Markers of inflammation. In: DeWitt JC , Rockwell CE , Bowman CC , eds. Immunotoxicity Testing: Methods and Protocols. Springer; 2018:57‐79. doi:10.1007/978-1-4939-8549-4_5 29882133

[4] Margraf A , Lowell CA , Zarbock A . Neutrophils in acute inflammation: current concepts and translational implications. Blood. 2022;139:2130‐2144. 34624098 10.1182/blood.2021012295 PMC9728535

[5] Jaillon S , Galdiero MR , del Prete D , Cassatella MA , Garlanda C , Mantovani A . Neutrophils in innate and adaptive immunity. Semin Immunopathol. 2013;35:377‐394. 23553214 10.1007/s00281-013-0374-8

[6] Matthay MA et al. Acute respiratory distress syndrome. Nat Rev Dis Primer. 2019;5:18. 10.1038/s41572-019-0069-0 PMC6709677 30872586

[7] Thompson BT , Chambers RC , Liu KD . Acute respiratory distress syndrome. N Engl J Med. 2017;377:562‐572. 28792873 10.1056/NEJMra1608077

[8] Auriemma CL , Zhuo H , Delucchi K , et al. Acute respiratory distress syndrome‐attributable mortality in critically ill patients with sepsis. Intensive Care Med. 2020;46:1222‐1231. 32206845 10.1007/s00134-020-06010-9 PMC7224051

[9] Hendrickson KW , Peltan ID , Brown SM . The epidemiology of acute respiratory distress syndrome before and after coronavirus disease 2019. Crit Care Clin. 2021;37:703‐716. 34548129 10.1016/j.ccc.2021.05.001 PMC8449138

[10] Villar J , Ferrando C , Martínez D , et al. Dexamethasone treatment for the acute respiratory distress syndrome: a multicentre, randomised controlled trial. Lancet Respir Med. 2020;8:267‐276. 32043986 10.1016/S2213-2600(19)30417-5

[11] Wang Q , Xie T , Gao R , et al. Neutrophil‐to‐lymphocyte ratio is a powerful predictor of adult patients with acute respiratory distress syndrome who might benefit from corticosteroid therapy. Am J Transl Res. 2021;13:11556‐11570. 34786082 PMC8581852

[12] Nicolaides NC , Chrousos G , Kino T . Glucocorticoid Receptor. In: Feingold KR et al., eds. Endotext. MDText.com, Inc.; 2000.

[13] Filep JG , Delalandre A , Payette Y , Földes‐Filep É . Glucocorticoid receptor regulates expression of L‐selectin and CD11/CD18 on human neutrophils. Circulation. 1997;96:295‐301. 9236448 10.1161/01.cir.96.1.295

[14] Rocksén D , Lilliehöök B , Larsson R , Johansson T , Bucht A . Differential anti‐inflammatory and anti‐oxidative effects of dexamethasone and N‐acetylcysteine in endotoxin‐induced lung inflammation. Clin Exp Immunol. 2000;122:249‐256. 11091282 10.1046/j.1365-2249.2000.01373.x PMC1905762

[15] Al‐Harbi NO et al. Dexamethasone attenuates LPS‐induced acute lung injury through inhibition of NF‐κB, COX‐2, and pro‐inflammatory mediators. Immunol Invest. 2016;45:349‐369. 27104958 10.3109/08820139.2016.1157814

[16] Mishler JM , Emerson PM . Development of neutrophilia by serially increasing doses of dexamethasone. Br J Haematol. 1977;36:249‐257. 871436 10.1111/j.1365-2141.1977.tb00646.x

[17] Aston WJ , Hope DE , Cook AM , et al. Dexamethasone differentially depletes tumour and peripheral blood lymphocytes and can impact the efficacy of chemotherapy/checkpoint blockade combination treatment. Onco Targets Ther. 2019;8:e1641390. 10.1080/2162402X.2019.1641390 PMC6791454 31646089

[18] Fromen CA , Kelley WJ , Fish MB , et al. Neutrophil–particle interactions in blood circulation drive particle clearance and Alter neutrophil responses in acute inflammation. ACS Nano. 2017;11:10797‐10807. 29028303 10.1021/acsnano.7b03190 PMC5709153

[19] Banka AL , Guevara MV , Brannon ER , et al. Cargo‐free particles divert neutrophil‐platelet aggregates to reduce thromboinflammation. Nat Commun. 2023;14:2462. 37117163 10.1038/s41467-023-37990-z PMC10144907

[20] Brannon ER , Guevara MV , Pacifici NJ , Lee JK , Lewis JS , Eniola‐Adefeso O . Polymeric particle‐based therapies for acute inflammatory diseases. Nat Rev Mater. 2022;7:796‐813. 35874960 10.1038/s41578-022-00458-5 PMC9295115

[21] Guevara MV et al. Rod‐shaped polymerized salicylic acid particles modulate neutrophil Transendothelial migration in acute inflammation. Adv Healthc Mater. 2025;14:2404955. 40348611 10.1002/adhm.202404955 PMC12184081

[22] Myerson JW , Patel PN , Rubey KM , et al. Supramolecular arrangement of protein in nanoparticle structures predicts nanoparticle tropism for neutrophils in acute lung inflammation. Nat Nanotechnol. 2022;17:86‐97. 34795440 10.1038/s41565-021-00997-y PMC8776575

[23] Brannon ER et al. Polysalicylic acid polymer microparticle decoys therapeutically treat acute respiratory distress syndrome. Adv Healthc Mater. 2022;11:1‐11. 10.1002/adhm.202101534 PMC8986552 34881524

[24] Brannon ER et al. Polymerized salicylic acid microparticles reduce the progression and formation of human neutrophil extracellular traps (NET)s. Adv Healthc Mater. 2025;14:2400443. 38898728 10.1002/adhm.202400443 PMC11628640

[25] Mathur R et al. Neutrophil hitchhiking enhances liposomal dexamethasone therapy of sepsis. ACS Nano. 2024;18:28866‐28880. 39393087 10.1021/acsnano.4c09054

[26] Lagreca E , Onesto V , di Natale C , la Manna S , Netti PA , Vecchione R . Recent advances in the formulation of PLGA microparticles for controlled drug delivery. Prog Biomater. 2020;9:153‐174. 33058072 10.1007/s40204-020-00139-y PMC7718366

[27] Sercombe L , Veerati T , Moheimani F , Wu SY , Sood AK , Hua S . Advances and challenges of liposome assisted drug delivery. Front Pharmacol. 2015;6:286. 26648870 10.3389/fphar.2015.00286 PMC4664963

[28] Safari H , Kaczorowski N , Felder ML , et al. Biodegradable, bile salt microparticles for localized fat dissolution. Sci Adv. 2020;6:eabd8019. 33277261 10.1126/sciadv.abd8019 PMC7821899

[29] Paskin A , Couasnon T , Perez JPH , et al. Nucleation and crystallization of ferrous phosphate hydrate via an amorphous intermediate. J Am Chem Soc. 2023;145:15137‐15151. 37409504 10.1021/jacs.3c01494 PMC10360157

[30] Bisso PW , Gaglione S , Guimarães PPG , Mitchell MJ , Langer R . Nanomaterial interactions with human neutrophils. ACS Biomater Sci Eng. 2018;4:4255‐4265. 31497639 10.1021/acsbiomaterials.8b01062 PMC6731026

[31] Saafane A , Girard D . Interaction between iron oxide nanoparticles (Fe3O_4_ NPs) and human neutrophils: evidence that Fe_3_O_4_ NPs possess some pro‐inflammatory activities. Chem Biol Interact. 2022;365:110053. 35872045 10.1016/j.cbi.2022.110053

[32] Jansch M , Stumpf P , Graf C , Rühl E , Müller RH . Adsorption kinetics of plasma proteins on ultrasmall superparamagnetic iron oxide (USPIO) nanoparticles. Int J Pharm. 2012;428:125‐133. 22342465 10.1016/j.ijpharm.2012.01.060

[33] Papini E , Tavano R , Mancin F . Opsonins and dysopsonins of nanoparticles: facts, concepts, and methodological guidelines. Front Immunol. 2020;11:567365. 33154748 10.3389/fimmu.2020.567365 PMC7587406

[34] Kalska‐Szostko B , Wykowska U , Satula D , Nordblad P . Thermal treatment of magnetite nanoparticles. Beil J Nanotechnol. 2015;6:1385‐1396. 10.3762/bjnano.6.143 PMC4505171 26199842

[35] Li XG , Chiba A , Takahashi S , Sato M . Oxidation characteristics and magnetic properties of iron ultrafine particles. J Appl Phys. 1998;83:3871‐3875.

[36] Zhao XQ , Liang Y , Hu ZQ , Liu BX . Oxidation characteristics and magnetic properties of iron carbide and iron ultrafine particles. J Appl Phys. 1996;80:5857‐5860.

[37] Meng X , Ryu J , Kim B , Ko S . Application of iron oxide as a pH‐dependent indicator for improving the nutritional quality. Clin Nutr Res. 2016;5:172‐179. 27482521 10.7762/cnr.2016.5.3.172 PMC4967720

[38] Domsta V , Boralewski T , Ulbricht M , Schick P , Krause J , Seidlitz A . Stability of dexamethasone during hot‐melt extrusion of filaments based on Eudragit® RS, ethyl cellulose and polyethylene oxide. Int J Pharm X. 2024;8:100263. 39040516 10.1016/j.ijpx.2024.100263 PMC11260382

[39] Santos WM , Nóbrega FP , Andrade JC , et al. Pharmaceutical compatibility of dexamethasone with excipients commonly used in solid oral dosage forms. J Therm Anal Calorim. 2021;145:361‐378.

[40] Oliveira PFM , Willart J‐F , Siepmann J , Siepmann F , Descamps M . Using milling to explore physical states: the amorphous and polymorphic forms of dexamethasone. Cryst Growth des. 2018;18:1748‐1757.

[41] Ummiti K , Vakkala S , Panuganti V , Annarapu MR . Isolation, identification, and characterization of 17‐Oxo dexamethasone, an oxidative degradation impurity of dexamethasone using flash chromatography and Nmr/Hrms/Ir. J Liq Chromatogr Relat Technol. 2014;37:2403‐2419.

[42] Patel P , Jamal Z , Ramphul K . Immunoglobulin. StatPearls. StatPearls Publishing; 2026. 30035936

[43] Kelley WJ , Fromen CA , Lopez‐Cazares G , Eniola‐Adefeso O . PEGylation of model drug carriers enhances phagocytosis by primary human neutrophils. Acta Biomater. 2018;79:283‐293. 30195083 10.1016/j.actbio.2018.09.001 PMC6181144

[44] Chen K , Zheng C , Lv Y , et al. Complement opsonized protein corona activated by precoated immunoglobulin enables neutrophil‐hitchhiking for rapid and enhanced drug delivery for acute liver failure recovery. Nano Today. 2024;59:102512.

[45] Mutua V , Gershwin LJ . A review of neutrophil extracellular traps (NETs) in disease: potential anti‐NETs therapeutics. Clin Rev Allergy Immunol. 2021;61:194‐211. 32740860 10.1007/s12016-020-08804-7 PMC7395212

[46] Vorobjeva NV , Chernyak BV . NETosis: molecular mechanisms, role in physiology and pathology. Biochem Mosc. 2020;85:1178‐1190. 10.1134/S0006297920100065 PMC7590568 33202203

[47] Dandona P , Aljada A , Ghanim H , Mohanty P , Hamouda W , al‐Haddad W . Acute suppressive effect of hydrocortisone on p47phox subunit of nicotinamide adenine dinucleotide phosphate oxidase. Metabolism. 2001;50:548‐552. 11319715 10.1053/meta.2001.22511

[48] Condino‐Neto A , Whitney C , Newburger PE . Dexamethasone but not indomethacin inhibits human phagocyte nicotinamide adenine dinucleotide phosphate oxidase activity by down‐regulating expression of genes encoding oxidase components1. J Immunol. 1998;161:4960‐4967. 9794432

[49] Grommes J , Soehnlein O . Contribution of neutrophils to acute lung injury. Mol Med. 2011;17:293‐307. 21046059 10.2119/molmed.2010.00138 PMC3060975

[50] Ericson‐Neilsen W , Kaye AD . Steroids: pharmacology, complications, and practice delivery issues. Ochsner J. 2014;14:203‐207. 24940130 PMC4052587

[51] Buchman AL . Side effects of corticosteroid therapy. J Clin Gastroenterol. 2001;33:289‐294. 11588541 10.1097/00004836-200110000-00006

[52] Walpole SC , Prieto‐Merino D , Edwards P , Cleland J , Stevens G , Roberts I . The weight of nations: an estimation of adult human biomass. BMC Public Health. 2012;12:439. 22709383 10.1186/1471-2458-12-439 PMC3408371

