# Supplementary material for: Iron‐dexamethasone nanoparticles mitigate acute neutrophilic inflammation
Source: Bioeng Transl Med. 2026 Aug 19:e70173. Online ahead of print. doi: 10.1002/btm2.70173 (PMC13490671; doi:10.1002/btm2.70173)
Supplement: Supplementary file 1 [file BTM2-9999-e70173-s001.docx]

**Figure S1: Dex NP Particle Associations with Human Neutrophils in Whole Blood.** This figure shows the two peaks associated with human neutrophils that have been treated with either PBS (blue) or FITC labeled Dex NP (pink).

**Figure S2: Surface Protein Corona Around Dex NP Heated to Various Temperatures.** SDS-Page was used to determine the total amount and type of proteins that formed protein coronas around Dex NP heated to either 200°C, 215°C, or 230°C after incubation in human serum.

**Figure S3: Physical Characterization of Dex NP and DexOx NP.** The XPS survey spectra of (**A**) Dex NP and (**B**) DexOx NP showed that iron, carbon, oxygen, fluorine, and phosphorus are contained within the particle matrix both before and after oxidation, as indicated by the Fe 2p, F 1s, C 1s, O 1s, and P 2p peaks, respectively; We used CasaXPS software to determine the Fe^2+^ and Fe^3+^ component peaks of the Fe 2p region of (**C**) Dex NP and (**D**) DexOx NP. The two peaks observed corresponded to Fe 2p_3/2_ and Fe 2p_1/2_ regions. The component peaks for Fe^2+^ were found at ~711.1 eV (Fe 2p_3/2_), ~724.6 eV (Fe 2p_1/2_), and ~718.0 eV (satellite). The component peaks for Fe^3+^ were found at ~713.5 eV (Fe 2p_3/2_), ~727.9 eV (Fe 2p_1/2_), and ~733.8 eV (satellite). The results suggest a mixed valence for iron contained within the particle, where a shift towards Fe^3+^ is observed for DexOx NPs that underwent open air oxidation; (**E**) FTIR spectra of Dex NP and DexOx NP; (**F**) XRD of Dex NP and DexOx NP.

**Figure S4: DexOx NP Degradation In PBS.** DexOx NP were degraded in PBS at 37°C for 8 hours, with samples taken at 1, 2, 4, and 8 hours. Samples were analyzed using a dexamethasone ELISA, which measures dexamethasone bioactivity.

**Figure S5: Dex NP and DexOx NP Safety Characterization in Whole Human Blood**. (**A**) Absorbance spectra of red blood cell supernatant after incubation with freely soluble dexamethasone phosphate, Dex NP, or DexOx NP; (**B**) Fold change of P-Selectin expressed on the surface of human platelets after incubation with dexamethasone phosphate, Dex NP, or DexOx NP, compared with platelets incubated with ADP as positive activation control. All data points represent individual human donors (n=5). One-Way ANOVA with post hoc Tukey’s test was used to determine statistical significance.

**Figure S6: Characterization of Human Neutrophil Activation with Dexamethasone Treatments.** (A) CD62L fold decrease is shown for human neutrophils in whole blood treated with dexamethasone treatments at a concentration of 0.1 mg/mL as compared to unactivated control; (B) CD62L fold decrease for human neutrophils in whole blood pre-incubated with mifepristone prior to addition of dexamethasone treatments, as compared to unactivated control. Each data point represents individual human donor (n=3). One-Way ANOVA with post hoc Fisher’s LSD was run for each experiment.

**Figure S7: Human Neutrophil Activation with Iron Oxide Nanoparticle Treatments**. (**A**) SEM image depicting commercially available iron (II) oxide nanoparticles that are approximately 100 nm in diameter; (**B**) SEM image depicting iron (III) oxide nanoparticles that are approximately 100 nm in diameter, made via heated oxidation from commercially purchased iron (II) oxide nanoparticles; (**C**) CD62L fold decrease is shown for human neutrophils in whole blood treated with iron oxide nanoparticle treatments at a concentration of 0.1 mg/mL as compared to unactivated control.

**Figure S8: NET Formation Following Incubation of Human Neutrophils with Dex NP.** (**A**) Comparison of NETs formed by unactivated human neutrophils as compared to human neutrophils treated with Dex NP; (**B**) Comparison of NETs formed by PMA activated human neutrophils as compared to PMA activated human neutrophils treated with Dex NP. All data points represent individual human donors (n=5). Data points connected via line are paired samples. Paired T-test was used for statistical analysis.

**Figure S9: In Vivo Safety of Dexamethasone Treatments**. (**A**) Blood plasma AST levels following treatment with dexamethasone phosphate, Dex NP, or DexOx NP at specified concentration after one week, unless otherwise specified; (**B**) and (**C**) depict total mouse neutrophil and monocyte counts, respectively, in whole blood one week following treatment, unless otherwise specified; (**D**) mouse weights over 7 days following treatment with specified dexamethasone treatments. All data points represent individual mouse samples (n=3). One-Way ANOVA was used to determine statistical significance, with no significance observed for any treatment group.

**Figure S10: Polystyrene Nanoparticle Associations with Mouse Neutrophils**. This figure shows the two peaks associated with mouse neutrophils that have been treated with either PBS (gray) or FITC labeled Dex NP (green).

**Figure S11: In Vivo Characterization of DexOx NP Behavior as Compared to Free Dexamethasone and Polystyrene NP**. (**A**) The percent neutrophils in whole blood for all treatment groups as compared to untreated control; (**B**) The total number of macrophages present with the BALF of LPS treated mice as compared to untreated control. Data points represent individual mice (n≥8). One-Way ANOVA with post hoc Fisher’s LSD used to determine statistical significance.

**Figure S12: Mouse BALF Cytokine and Chemokine Analysis**. Total concentration of (**A**) IL-6, (**B**) TNF-α, (**C**) KC, and (**D**) MIP-2 in mouse BALF samples following LPS instillation and specified treatments. Each data point represents individual mouse (n≥8). One-Way ANOVA with post hoc Fisher’s LSD used to determine statistical significance.
